# Diode Laser Photocoagulation of Oral Venous Malformations in Patients on Anticoagulant Therapy Without Drug Discontinuation

**DOI:** 10.7759/cureus.7340

**Published:** 2020-03-20

**Authors:** Fabio Dell'Olio, Domenico De Falco, Simona Di Nanna, Assunta Casorelli, Gianfranco Favia

**Affiliations:** 1 Interdisciplinary Department of Medicine, University of Bari Aldo Moro, Bari, ITA; 2 Dentistry, University of Bari Aldo Moro, Bari, ITA

**Keywords:** diode laser, venous malformations, photocoagulation, anticoagulant therapy, oral surgery

## Abstract

The diode laser is widely used for the treatment of venous malformations of the oral cavity nowadays. Anticoagulant therapy is usually modified or suspended in patients needing oral surgery, especially for vascular lesion treatment. We report a case series of venous malformations in patients on anticoagulant therapy treated by diode laser photocoagulation without drug discontinuation.

## Introduction

Vascular anomalies (VAs) arise from blood vessel abnormalities or during endothelial proliferation and are classically distinguished according to the classifications of Mulliken and Glowacki, based on their clinical, histological, and histochemical findings, in hemangiomas and vascular malformations (VMs) [1-2]. The differences among VAs have been widely debated and revisited over the years. Recently, VAs have been reanalyzed by the International Society for the Study of Vascular Anomalies (ISSVA), which classified them in vascular tumors (benign, locally aggressive or borderline, and malignant) and VMs [3-4]. The latter are subclassified as follows: (a) simple; (b) combined, when two or more vascular malformation types are found in a single lesion; (c) anomalies of major vessels, also known as "channel type" or "truncal" VMs; anomalies of (d) origin, (e) course, (f) number, (g) length, (h) diameter; the last group also includes syndromic VMs associated with other anomalies. Diode lasers are generally accepted as effective medical devices to treat VMs in the head and neck as providing a targeted selectivity for oxyhemoglobin, induction of photothermolysis and erythrocyte micro-agglutination and vessel obliteration [5-9]. Anticoagulant therapy is usually modified or suspended in patients needing oral surgery procedures to prevent intra- and postoperative bleeding, especially for vascular lesions treatment. We report on a case series of VMs occurring in patients on oral anticoagulant therapy (warfarin) treated by diode laser photocoagulation without drug discontinuation [10-11].

## Case presentation

Case 1

**Figure 1:**
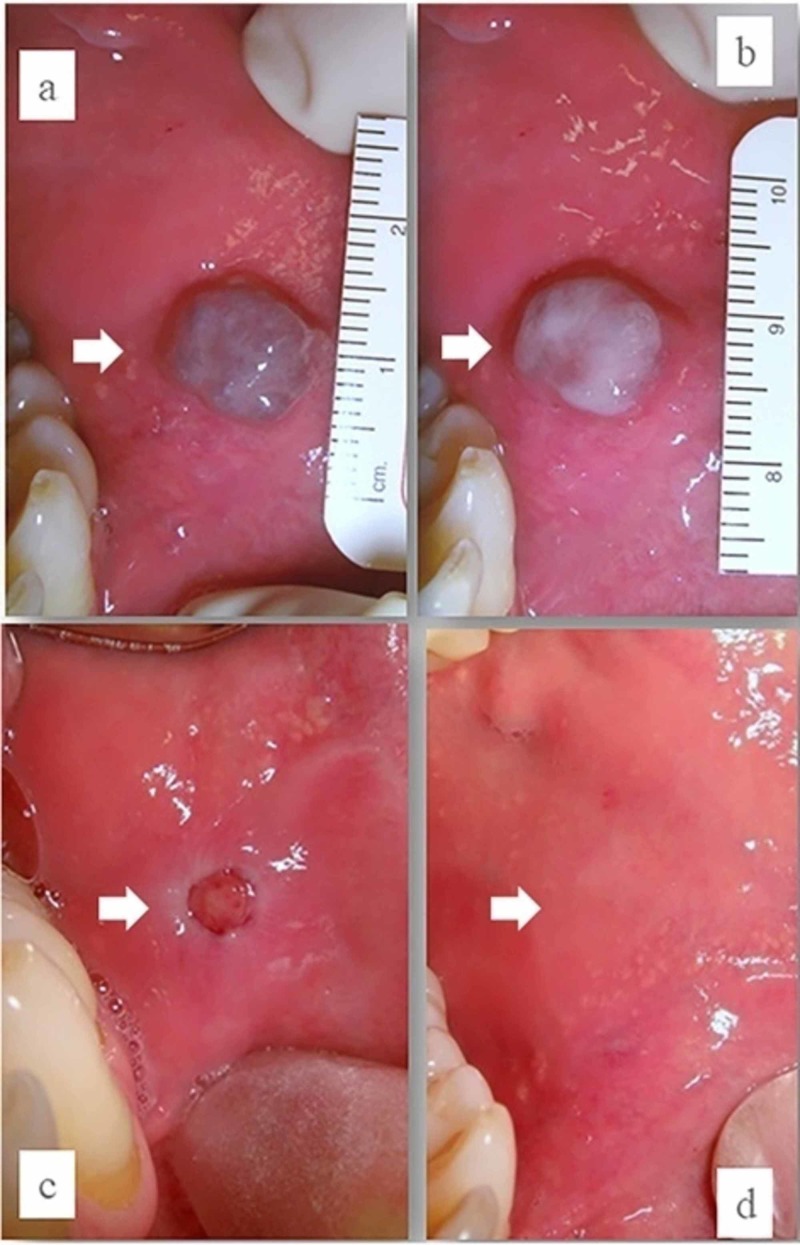
Slowly growing violet-blue lesion of the left cheek of two years duration in a 60-year-old male (A) blue-violet appearance of venous malformation of the cheek; (B) color variation to white-grey after diode laser treatment; (C) clinical appearance after 12 days; (D) complete healing after 18 days

A slowly growing, violet-blue lesion of the left cheek of two years duration was seen in a 60-year-old male (Figure 1A). The patient’s medical history revealed that he was suffering from long-lasting, chronic atrial fibrillation, treated with warfarin in order to prevent thromboembolic complications; he reported suffering from dental anxiety too. With the clinical diagnosis of VM, photocoagulation with diode laser was proposed to all patients without drug discontinuation. Under light conscious sedation and with local infiltration of anesthesia, the lesion was photocoagulated by diode laser (wavelength 800 ± 10 nm; continuous wave, output energy 5W). The treatment ended when color variation from blue-violet to white-grey was observable (Figure 1B). No intra- and postoperative bleeding was observable. The patient was followed-up after 10 days (Figure 1C) and completely healed after 18 days (Figure 1D).

Case 2

**Figure 2:**
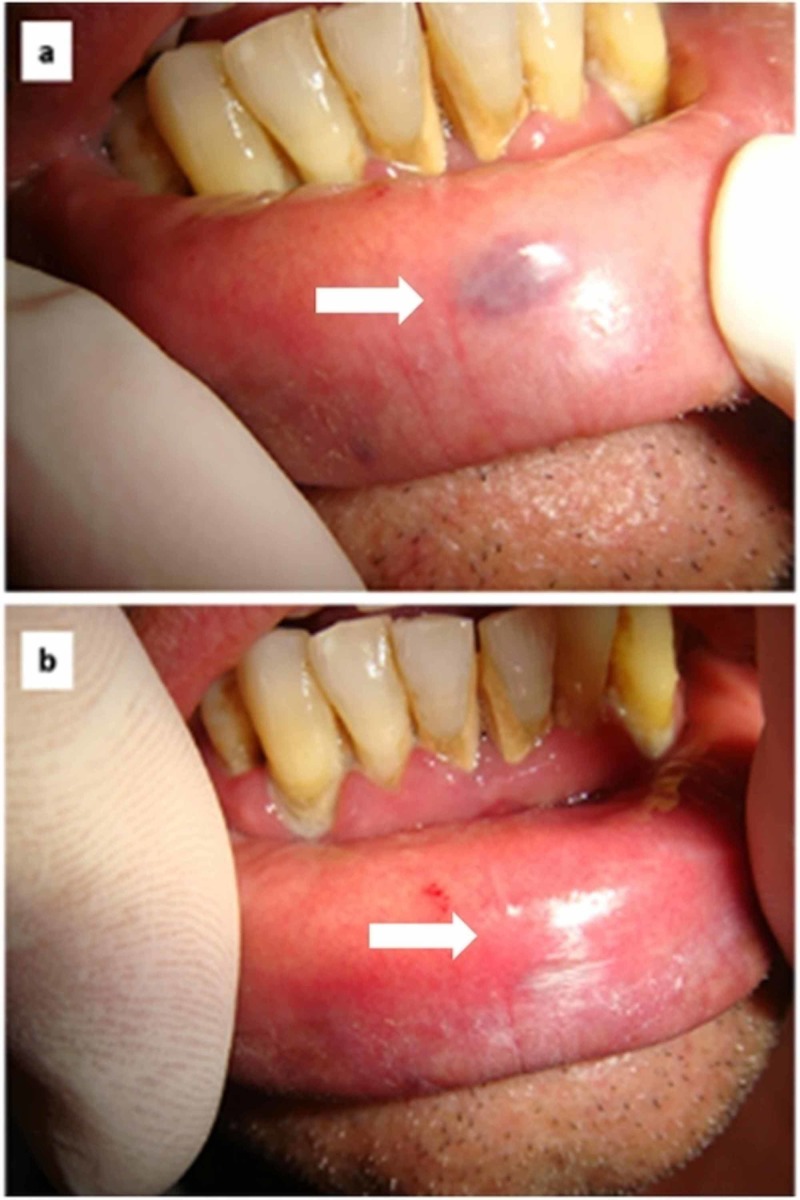
Rapidly growing bluish lesion of the lip vermilion in a 55-year-old male (A) small bluish lesion of the lip vermilion; (B) complete healing 12 days after photocoagulation by diode laser

A rapidly growing bluish lesion of the lip vermilion was seen in a 55-year-old male receiving warfarin for the prevention of a thromboembolic event following the implantation of a mechanical heart valve (Figure 2A). The lesion was small but deep into the underlined tissue with a rather thick covering mucosa. With local infiltration of anesthesia, the lesion was photocoagulated by diode laser (wavelength 800 ± 10 nm; continuous wave, output energy 3W) without warfarin discontinuation till the color variation from blue to white-grey. No intra- and postoperative bleeding was observable. Complete mucosal healing was observed after 12 days (Figure 2B) without cosmetic complications.

Case 3

**Figure 3:**
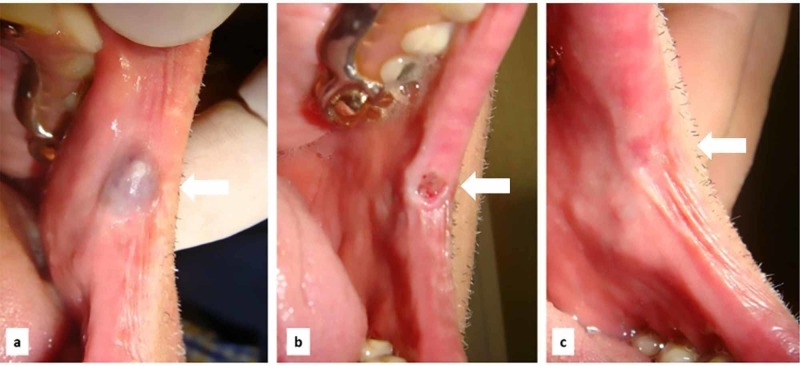
Slowly growing blue-violet lesion of the commissura labialis oris in a 64-year-old male (A) venous malformation of the commissura labialis; (B) 10 days after diode laser photocoagulation; (C) complete resolution after 16 days

A slowly growing blue-violet lesion of the commissura labialis oris was seen in a 64-year-old male patient affected by hypertension and atrial fibrillation, in therapy with antihypertensive drug and warfarin to prevent thromboembolic complications (Figure 3A). The patient reported recurrent chewing trauma of the area related to the incongruous removable prosthesis. The lesion was photocoagulated by diode laser (wavelength 800 ± 10 nm; continuous wave, output energy 5W) without drug discontinuation; bleeding was totally absent during the procedure as well as the postsurgical edema. The patient was followed up after 10 days (Figure 3B) and completely healed after 16 days (Figure 3C).

## Discussion

The advantages of diode laser application in oral surgery are the lack of bleeding during cutting, reduction of postoperative edema, unnecessary stitches, fast mucosal healing [5-6,8-9]. For such reasons, the introduction of laser therapy in the last two decades represented a significant innovation in several oral surgery procedures, e.g., periodontal decontamination, gingival overgrowth, and surgical excisions of benign and malignant neoplasms [5,7,12-13]. The use of diode laser to treat oral VMs and VLs is associated with a shorter operating time and fewer postoperative complications as compared to the conventional scalpel surgery. Nevertheless, more than one session may be required for larger and deeper VMs [5,9]. Overall, diode laser treatments have multiple benefits, being non-invasive, conservative, and repeatable as needed, e.g., to treat larger and/or deeper lesions [5-6]. In addition, unnecessary drug suspension when the patient is on anticoagulant therapy (e.g., warfarin), as well as the use of conscious sedation for the treatment of patients suffering dental anxiety, as in one of the reported cases, makes such treatment even less invasive [10-11,14]. Conceptually, the absence of intra-operative bleeding also reduces the risk of cross-infection during (surgical and not surgical) dental procedures in patients with infectious disease (hepatitis C virus (HCV), hepatitis B virus (HBV), human immunodeficiency virus (HIV)), thus leading to a simplified approach toward these patients [15-17].

## Conclusions

Among all lasers with proven surgical capabilities, the diode laser is widely used for the surgical excision of proliferating lesions and the photocoagulation of small and large VMs in the oral cavity. The main problem in such patients, especially when large and/or in therapy with anticoagulants, is the prevention of hemorrhagic complications during treatment and in the immediate postoperative course. Along with the good clinical outcomes demonstrated with this report, the diode laser greatly simplifies treatment for the clinician, avoiding the preoperative assessment/modification of therapy, preoperative computed tomography (CT) angiography, general anesthesia, prolonged hospitalization, potential life-threatening hemorrhagic complications, recurrence, and, consequently, increasing the acceptability by patients. Anticoagulant therapy may safely be not discontinued for small VM treatment, as in the reported cases. Further studies will be needed to confirm it even in the cases of larger VMs in the head and neck.
